# Advances in Endoscopic Diagnosis and Management of Cholangiocarcinoma

**DOI:** 10.3390/jcm14176028

**Published:** 2025-08-26

**Authors:** Usamah Chaudhary, Shawn L. Shah

**Affiliations:** 1 Department of Internal Medicine, University of Texas Southwestern Medical Center, Dallas, TX 75390, USA; usamah.chaudhary@utsouthwestern.edu; 2 Division of Digestive and Liver Diseases, Dallas VA Medical Center—VA North Texas Healthcare System and University of Texas Southwestern Medical Center, Dallas, TX 75390, USA

**Keywords:** biliary tract cancer, cholangiocarcinoma, radiofrequency ablation, photodynamic therapy, advanced endoscopy

## Abstract

Cholangiocarcinoma (CCA) is an aggressive malignancy originating from the epithelial lining of the intrahepatic or extrahepatic bile ducts. Although rare globally, its mortality closely mirrors incidence due to late-stage presentation of the disease and limited curative options. While surgical resection and liver transplantation remain the cornerstone treatments for those with resectable disease, endoscopic techniques have emerged as versatile tools for diagnosis, therapy, and palliation. In recent years, there have been major advancements in endoscopic therapies, including radiofrequency ablation (RFA), intraluminal brachytherapy (ILBT), and photodynamic therapy (PDT). The current narrative review serves to provide an overview of current and emerging endoscopic strategies for CCA, emphasizing diagnostic capabilities, therapeutic approaches, palliative interventions, and future directions.

## 1. Introduction

Biliary tract cancers comprise malignancies arising from the gallbladder, ampulla of Vater, and bile duct. Cholangiocarcinomas (CCAs) are epithelial cell malignancies that can occur within the biliary tract and/or hepatic parenchyma. These are heterogeneous cancers and are further classified based on their histologic and anatomic involvement. CCAs can be broadly characterized as mass-forming, periductal-infiltrating, or intraductal-growing as well as intrahepatic CCA (iCCA), perihilar CCA (pCCA, also known as Klatskin tumors), or distal CCA (dCCA). Distal CCA involves tumor within the common bile duct (CBD) below the cystic duct, whereas pCCA involves tumor between the cystic duct insertion and the second-order bile ductules. Tumor proximal to the second-order bile ductules within the liver is defined as iCCA. Precise anatomical and histological classification of CCA is paramount as it influences both clinical presentation and therapeutic approach.

### 1.1. Epidemiology

CCAs are rare cancers worldwide and highly lethal due to their late-stage diagnosis and limited treatment options. The global annual incidence of CCA ranges from 0.35–2 per 100,000 individuals, with mortality closely paralleling incidence due to the aggressive nature of the disease [1]. The epidemiology of CCA varies globally, reflecting regional differences in risk factors. For instance, the incidence rates of CCA are significantly higher in Southeast Asia with worldwide age-standardized rates highest in Thailand due in large part to the increased rates of liver fluke infections. Whereas in Western countries, the majority of cases are associated with primary sclerosing cholangitis (PSC) [2,3]. In general, CCA tends to affect predominantly males and older individuals. While extrahepatic CCA remains more prevalent, the incidence of iCCA has risen disproportionately over recent decades [4,5,6]. However, the true incidence of the different subtypes of CCA is unclear due to misclassification in national databases and mischaracterization of pCCA and dCCA as extrahepatic CCA rather than distinct entities [2].

### 1.2. Risk Factors

Established risk factors for CCA include chronic biliary inflammation due to PSC, chronic liver diseases (e.g., cirrhosis and viral hepatitis B and C), liver fluke infections, hepatolithiasis, and bile duct cysts [1,7,8,9]. Metabolic conditions such as diabetes, thyrotoxicosis, and chronic pancreatitis have also been implicated in CCA. Different risk factors often predict subtypes of CCA; for example, chronic liver diseases are more often associated with iCCA, while chronic pancreatitis and choledocholithiasis are more associated with dCCA. Environmental and lifestyle factors, including chronic inflammation, obesity, and smoking, have also been implicated in CCA pathogenesis [10]. A systematic review and meta-analysis found the strongest risk factors to be biliary cysts and stones, cirrhosis, and viral hepatitis B and C [9].

Parasitic liver fluke infections (e.g., Opisthorchis viverrini, Clonorchis sinensis, and Opisthorchis felineus) are endemic to Southeast Asia and constitute major risk factors for CCA in these regions [11]. Transmission commonly occurs through consumption of undercooked cyprinid fish, resulting in infection as the fish is a carrier of the larval parasite. In liver fluke infection, the parasitic worm migrates and feeds throughout the biliary tract. This in turn causes chronic biliary inflammation through mechanical injury and granulomatous response, ultimately promoting carcinogenesis via cytokine-mediated fibrosis [12,13].

In the Western world, PSC is the largest risk factor for CCA development. CCA can develop at any point in the disease process of PSC; however, the incidence (up to 50%) is highest within the first year of diagnosis. The pathogenesis remains incompletely understood, but it is likely due to the inflammatory response (e.g., within dominant strictures) and genetic changes. A large population-based cohort study in the Netherlands (*n* = 590 patients with PSC) found that the cumulative risk for developing CCA was 6%, 14%, and 20% at 10, 20, and 30 years after PSC diagnosis, respectively, which is significantly greater than that of the general population [14,15,16,17,18]. According to the American Association for the Study of Liver Diseases (AASLD) guidelines, patients with PSC should undergo yearly screening for CCA with magnetic resonance imaging (MRI) and magnetic resonance cholangiopancreatography (MRCP) along with serum levels of carbohydrate antigen 19-9 (CA 19-9) [18].

### 1.3. Diagnosis

The diagnosis of CCA can be challenging given its often indolent and non-specific symptoms. With the ubiquitous use of cross-sectional abdominal imaging, many cases of CCA, particularly iCCA, are often found incidentally on imaging. The clinical presentation of CCAs varies widely depending on the anatomic location of the cancer. CCAs often present with non-specific symptoms, such as fever, abdominal pain, and/or weight loss, leading to delayed diagnosis and, as a result, poor prognosis. Furthermore, patients with extrahepatic CCA are more likely to present with jaundice due to an obstructing intrahepatic mass within the common bile or hepatic duct.

The workup for suspected CCA should include multiphasic contrast-enhanced computed tomography (CT) imaging or MRI with MRCP to characterize ductal anatomy with tumor involvement, assess lesion vascularity, and distinguish primary hepatobiliary cancers from metastatic disease [19]. A multidisciplinary team and tumor board should review these cases prior to any invasive intervention, as even minor interventions can lead to inflammatory changes that may underestimate or overestimate tumor burden and involvement [20,21]. Endoscopic ultrasound (EUS) and endoscopic retrograde cholangiopancreatography (ERCP) have become indispensable tools in the management of cholangiocarcinoma and its associated sequelae, though their optimal use remains incompletely characterized per contemporary guidelines. Here, we aim to review the role of endoscopy as a diagnostic, therapeutic, and palliative instrument for CCA.

## 2. Endoscopic Methods for Diagnosis

CCAs tend to grow in a longitudinal fashion along the bile duct, making imaging studies such as MRI or CT often non-diagnostic [22,23]. In these cases, direct visualization or tissue sampling via various endoscopic techniques is often required for diagnosis. The National Comprehensive Cancer Network (NCCN) guidelines recommend tissue acquisition only be performed in patients with suspected CCA after determination of transplant status and resectability candidacy [4].

### 2.1. Endoscopic Retrograde Cholangiopancreatography (ERCP)

ERCP offers tremendous diagnostic and interventional capabilities for patients with CCA. The NCCN guidelines suggest a preference for MRCP over ERCP for initial diagnosis and pre-operative staging of pCCA given improved sensitivity, specificity, and diagnostic accuracy. As for extrahepatic CCA diagnosis, the guidelines recommend avoidance of ERCP to avoid biliary tract contamination and/or complications. However, ERCP should be considered in patients with unresectable disease requiring diagnosis or in patients in whom a therapeutic or palliative intervention is warranted [4]. The choice for ERCP tends to be institution specific after multidisciplinary discussion.

ERCP offers several sampling methods for suspected CCA including bile aspiration, brush cytology, and intraductal biopsy. Historically, ERCP-guided brush cytology was the standard for tissue acquisition given the ease of performing and high specificity. However, numerous studies and meta-analyses have shown that brush cytology alone has limited sensitivity (Table 1). A large meta-analysis of ERCP-guided brush cytology (*n* = 1123) found a sensitivity of 56% compared to 67% for ERCP-guided biopsy (*n* = 719). When combined, ERCP-guided brush cytology and biopsy together (*n* = 358) yielded a sensitivity of 70.7% [24]. Another meta-analysis of nine studies found similar diagnostic rates between brushings and biopsies, with modest improvement in sensitivity when the diagnostic approaches were combined (59.4%, 95% CI, 53.7–64.8%) [25].

The emergence of high-definition cholangioscopy has allowed for direct visualization of biliary tract lesions and targeted biopsies. Multiple meta-analyses of cholangioscopy-guided biopsies have found improved diagnostic accuracy compared to ERCP-guided brush cytology and biopsy with wide variation in sensitivity and specificity [38,39,40]. This range in sensitivity may be attributable to the difficulty of performing cholangioscopy in these patients, and further studies specifically with patients with CCA are needed, rather than cohorts of patients with indeterminate bile duct strictures. Imaging-based classification systems such as the Monaco classification can be used during ERCP to differentiate between benign and malignant strictures. However, even in expert hands there remains moderate interobserver variability of optical features from cholangioscopy, making sampling critical for diagnosis [41]. The diagnostic yields of the various tissue acquisition methods for CCA are summarized in Table 1.

Advances in tissue analysis methods such as fluorescence in situ hybridization (FISH)—technology to detect genetic aberrations in DNA sequence—have led to further improvements in sensitivity and specificity of CCA tissue samples [42,43]. In a retrospective study of 92 patients (*n* = 41 with CCA), the addition of brush cytology with FISH with cholangioscopy-guided biopsy increased the sensitivity from 44.7% with brush cytology alone to 71.4% (*p* = 0.03) for the detection of malignancy. However, the combination of these modalities did not improve the sensitivity for detection of malignancy in patients with PSC [44]. Furthermore, patients with polysomy detected by FISH or serial polysomy results are at higher risk of developing CCA than those with non-polysomy (e.g., trisomy or tetrasomy) and a one-time polysomy result, respectively [45,46].

Molecular profiling using next-generation sequencing (NGS) has emerged as an important technique in improving the diagnosis of CCA. Recent studies have shown that incorporating NGS with ERCP-guided samples improves diagnostic yield [47,48]. Gene variants detected by NGS have been shown to be particularly useful as actionable treatment targets in up to 50% of patients [49]. Similarly, cancer-associated fibroblasts that comprise the stroma of CCA express various factors that have been studied in tumor progression, growth rates, and survival and have correlated with outcomes in CCA. These include factors such as α-smooth muscle actin (α-SMA), platelet-derived growth factor D (PDGF-D), and various interleukins [50,51,52,53,54]. These markers have major impacts on the prognosis and treatment planning for CCA. As genomic analysis further develops, detecting the expression levels of these molecular markers will become increasingly important.

### 2.2. Endoscopic Ultrasound (EUS)

EUS provides high-resolution imaging of the biliary tract and supports both diagnostic and therapeutic interventions. On EUS, typical CCA features include an intraductal mass invading the bile duct epithelium, periductal infiltration, or bile duct wall thickness greater than 3 mm [33,55]. Contemporary NCCN guidelines suggest that EUS can be utilized to establish tissue diagnosis in those who present with biliary obstruction or abnormality on cross-sectional abdominal imaging after surgical consultation and determination of transplant status. A prospective study of 228 patients with biliary strictures (*n* = 81 with CCA) who underwent EUS-guided fine needle aspiration (FNA) found the sensitivity for CCA to be to 73% (95% CI, 62–82%), with greater accuracy for dCCA compared to proximal CCA (81% vs. 59%, respectively, *p* = 0.04). The study also found EUS to be superior to MRI and CT in the diagnosis of CCA (94% vs. 42% and 30%, respectively) as smaller lesions can often be missed [33,56]. A meta-analysis comparing EUS and ERCP sampling modalities (i.e., ERCP brushings, ERCP biopsies, ERCP brushings and biopsies, and EUS-FNA) found EUS-FNA to have the highest sensitivity (73.6%, 95% CI, 64.7–81.5%) with equivocal rates of adverse events among the various sampling techniques [24]. EUS-FNA is particularly valuable in cases with non-diagnostic biopsy results from other prior sampling methods; however, optimization and standardization of adequate tissue acquisition with EUS continue to evolve [32]. The American Society of Gastrointestinal Endoscopy (ASGE) recommends use of EUS-FNA in addition to ERCP in three instances for indeterminate biliary strictures: (1) prior ERCP with non-diagnostic results, (2) distal biliary stricture, or (3) presence of lymphadenopathy or metastatic disease on cross-sectional imaging [57]. In addition to tumor staging, EUS can also serve as a valuable tool in lymph node staging. EUS-FNA has demonstrated clinical benefit in identifying malignant regional lymph nodes, accurately identifying malignancy in 87.1% of patients. EUS has also been shown to be superior to cross-sectional imaging at locating regional lymph nodes (86% vs. 47%, *p* < 0.001) [58].

Despite advances in EUS tissue acquisition, there continues to remain concern for seeding and/or contamination of the peritoneum with malignant cells from FNA. However, compared to percutaneous FNA where peritoneal seeding is not infrequent [59], EUS-FNA is often performed through the duodenal bulb in the retroperitoneum, an area that is typically surgically resected for dCCA [33]. Nonetheless, at present, the NCCN guidelines recommend tissue acquisition in patients with unresectable or metastatic disease and surgical exploration with consideration of laparoscopic staging for those with resectable disease. A retrospective study analyzing the risk of pre-operative EUS-FNA in patients with CCA (*n* = 150 with CCA, *n* = 61 with pre-operative EUS-FNA) found no increase in adverse events or effect on survival [60]. A large meta-analysis (*n* = 13,238) of patients with pancreatic lesions found there to be no risk of peritoneal dissemination with EUS-FNA [61]. Additionally, a multicenter retrospective study (*n* = 170) of patients undergoing EUS-FNA for gallbladder cancer found no cases of needle tract seeding [62]. Although the risks of needle tract seeding are exceedingly low, this should be discussed with the patient and, as previously mentioned, only be considered after the patient is not considered a surgical or transplant candidate. This continues to remain an area of much interest and, at present, needs to be individualized after multidisciplinary discussions.

### 2.3. Intraductal Ultrasonography (IDUS)

Intraductal ultrasonography (IDUS) is an alternate imaging modality for pancreaticobiliary diseases that uses an ultrathin caliber ultrasound probe that is introduced through the duodenoscope and up the bile duct. The addition of IDUS to ERCP-guided tissue sampling in a study of 60 patients with biliary strictures increased sensitivity from 48% to 90% and negative predictive value from 64% to 90% [63]. As compared with ERCP tissue sampling, IDUS, when combined with optical visualization, demonstrated superior sensitivity (89% (95% CI, 67–97%) vs. 83% (95% CI, 60–94%)) and specificity (92% (95% CI, 64–98%) vs. 42% (95% CI, 19–67%)) [64]. IDUS can help stage CCA with the ability to visualize the longitudinal tumor extent as well as extent of tumor invasion through the bile duct. A longitudinal study enrolling patients from 1989 to 2002 found IDUS to have excellent diagnostic accuracy for determining depth of tumor invasion (84.6%), pancreatic invasion (88.9%), and portal vein invasion (92.3%) [65]. However, use of IDUS still remains limited due to lack of widespread availability and need for specialized equipment.

### 2.4. Probe-Based Confocal Laser Endomicroscopy (pCLE)

Probe-based confocal laser endomicroscopy (pCLE) is a novel endoscopic modality that uses a fiber-optic probe through the duodenoscope, providing in vivo microscopic cellular and subcellular examination of the biliary epithelium. After intravenous injection of fluorescein, the probe provides high-resolution images of the mucosa and senses tissue illumination with a low-power laser to detect fluorescein reflection in target areas [7]. A study evaluating pCLE in the diagnosis of CCA (*n* = 14) found improved sensitivity and specificity compared to ERCP-guided sampling (83% vs. 79% and 88% vs. 50%, respectively). The study found specific patterns of neovascularization in CCA not found in benign strictures, which included large fluorescein-filled tortuous, dilated, and saccular vessels with inconsistent branching. This pattern likely correlates with the angiogenesis associated with CCA development [66]. In 2011, an initial expert consensus, called the Miami classification, was proposed for pCLE findings associated with malignancy and was further refined in 2013 with the Paris classification [67,68]. In the updated classification, bile ducts are classified as normal, inflammatory strictures, or malignant strictures based on pCLE findings. Malignant features identified via pCLE include thick dark bands (>40 µm) of collagen fibrils, thick white bands (>20 µm) of vessels, fluorescein leakage, visualization of villi or glands, and background dark clumps. Multiple meta-analyses of pCLE have shown high diagnostic ability in differentiating malignant from benign strictures [69,70,71,72]. Although early results are promising, pCLE remains limited in its widespread adoption due to high cost, limited availability, high interuser variability, and a significant learning curve. Further studies and standardization methods building on the Miami and Paris classification systems are needed before pCLE becomes a mainstay in the diagnostic algorithm for CCA.

## 3. Endoscopic Methods for Management

Endoscopic management of CCA is pursued after the disease has been determined to not be amenable to surgical intervention or liver transplantation. Historically, endoscopic approaches were largely palliative in nature; however, recent years have seen significant advances in endoscopic therapeutic offerings to patients with a diagnosis of unresectable or metastatic CCA. Endoscopic management ranges from relieving biliary obstruction with endoprosthesis placement to locoregional therapies with photodynamic therapy (PDT), radiofrequency ablation (RFA), and intraductal brachytherapy (ILBT). A brief overview of endoscopic locoregional therapies is provided in Table 2. Increasing data have demonstrated that these less invasive therapies improve quality of life and delay CCA-associated sequelae such as obstructive jaundice, cholangitis, and liver failure.

### 3.1. Biliary Drainage

The most common CCA-related sequela is biliary obstruction, leading to jaundice and ascending cholangitis [73]. Traditionally, biliary decompression has been achieved with ERCP, however EUS-guided biliary drainage (EUS-BD) has also emerged as a technique in the appropriate patient population [74,75,76]. Per contemporary NCCN guidelines, the route of biliary drainage when indicated in patients with potentially resectable disease should align with institutional expertise, targeting drainage of segments that are opacified and ideally >50% of viable volume [4].

EUS-BD has emerged as a viable option for biliary decompression in patients with unresectable disease and an inaccessible papilla. In recent years, EUS-BD has increasingly become a first-line therapy rather than rescue therapy for malignant distal biliary obstruction at expert centers. Dilation of the bile duct (typically at least 12 mm) is critical for the success of EUS-BD. EUS allows for multiple access points along the biliary tract with the most common being extrahepatic transluminal drainage via choledochoduodenostomy (in patients with dCCA), while less common is intrahepatic transluminal drainage via hepaticogastrostomy (in patients with pCCA). In a systematic review and meta-analysis of six randomized controlled trials comparing EUS-BD to ERCP-guided biliary drainage for patients with malignant biliary obstruction, the EUS approach was associated with significantly lower risk of reintervention, post-procedure pancreatitis, tumor ingrowth/overgrowth, and decreased hospital stay with no difference in safety and efficacy [77]. In a meta-analysis of 23 studies (*n* = 1437 cases), EUS-BD with lumen-apposing metal stents (LAMSs) had a technical success rate of 91.5% (95% CI 87.7–94.2%) and a clinical success rate of 87% (95% CI 82.3–90.6%) [78]. The positioning of the EUS approach as compared to ERCP remains debated; however, given the technical and technological limitations with EUS-BD, ERCP still remains the mainstay approach at present [79].

Biliary stent placement can be performed with plastic stents or self-expandable metal stents (SEMSs) tailored to unique scenarios. In particular for patients with unresectable pCCA, a randomized controlled trial found that metal stents provide more durable drainage and improved survival compared to placement of plastic stents [80]. Furthermore, a large retrospective study (*n* = 1989) comparing plastic stents to SEMSs in the management of malignant biliary obstruction found SEMSs to have lower occlusion rates, less need for reintervention, and lower rates of cholangitis [81]. In large part, this is attributable to the larger diameter of metal stents.

The number of stents to place has been debated over recent years due to mixed results with some studies suggesting benefit to bilateral stent placement, while other studies finding no significant difference in outcomes [81,82,83,84]. Part of the variability in results is attributable to the fact that bilateral stent placement can be technically challenging given the severity of the CCA-related stricture. Traditional techniques include stent-in-stent and side-by-side stent placement [85,86]. However, there have been many advances in novel stent designs such as stents with a more patent central mesh to make it easier for the contralateral stent to pass through, Y-shaped stents, and pre-deployment systems to allow for simultaneous side-by-side stent placement [87,88,89,90]. A large retrospective review (*n* = 480) found the patency of bilateral stents to be superior to that of unilateral stent placement irrespective of plastic or metal subtype [91]. As well, an open-label, randomized trial from Japan found no significant difference between suprapapillary plastic versus uncovered metal stent placement (with ability to place multiple stents) in patients with unresectable malignant hilar obstruction. Ultimately, allowing for drainage of liver segments that opacify with cholangiography promotes more favorable outcomes [92]. For patients with resectable CCA, endoscopic stent placement is typically reserved for those presenting with cholangitis, jaundice, or when surgical management is delayed. Most patients with dCCA proceed directly to pancreaticoduodenectomy, and endoscopic stent placement for biliary drainage typically does not provide any added benefit pre-operatively [93]. For operable pCCA, there has been much debate regarding use of preoperative biliary drainage (PBD) given conflicting results on efficacy and increased risk of infection [94,95]. A large meta-analysis (*n* = 3059) found pre-operative biliary drainage (PBD) to carry higher risks of long-term follow-up mortality, morbidity, post-operative infection, among numerous other complications. This study found PBD only to be beneficial in patients with an initial bilirubin above 218.75 μmol/L, portal vein embolization, and malnutrition [96]. One study of PBD found improved outcomes when the future liver remnant (FLR) after surgical resection was less than 30%, but no improvement if the FLR was greater than 30% [94]. PBD in Asian countries is routinely performed prior to surgical intervention [97,98,99]; however, in Western countries, there remains less widespread adoption due to concern for increased risk of infection with the endoscopic approach and possibility of metastatic seeding with the percutaneous approach [100,101]. The British Society of Gastroenterology (BSG) recommends a case-by-case multidisciplinary discussion regarding PBD and which modality to pursue among the options of percutaneous transhepatic biliary drainage, endoscopic nasobiliary drainage, and endoscopic biliary stenting. The guidelines comment that studies have shown increased rates of portal vein injury, catheter tract recurrence, and peritoneal dissemination with the percutaneous approach in addition to a randomized controlled trial that was halted due to the high complication rate in the percutaneous group [102]. The guidelines also suggest a bilirubin cutoff of 14.5 mg/dL to pursue stent placement [21].

### 3.2. Photodynamic Therapy (PDT)

PDT is an ablative therapy that involves the intravenous systemic administration of a photosensitizer for 48–72 h prior to performing ERCP. This photosensitizing material is preferentially taken up by dysplastic and neoplastic cells in the biliary tract. During ERCP, a specialized scope with a laser fiber is passed through the duodenoscope and applies selective irradiation with an activating light, actuating the photosensitizer and releasing oxygen free radicals with resultant tissue necrosis and apoptosis. PDT use in CCA was first described in 1991 [103], and many studies since have shown PDT as a promising adjunctive therapeutic modality for patients with unresectable CCA.

Multiple meta-analyses have shown that, in combination with stenting or chemotherapy, PDT improves overall survival and quality of life in patients with unresectable CCA without increasing adverse events [104,105,106,107]. A prospective study of 184 patients with pCCA comparing outcomes in those who underwent surgical resection (borderline resectable CCA), PDT with stenting, and stenting alone found that PDT with stenting had equivalent outcomes with R1 (microscopic infiltration of the resection and/or dissection margins) and R2 (gross residual disease) resections and had improved outcomes as compared to stenting alone [108]. Numerous subsequent studies on the outcomes of PDT as compared to stenting have found that PDT results in longer survival. Additionally, shorter time between diagnosis and PDT was also associated with survival benefits [109,110]. PDT prior to stent placement has also been shown to prolong metal stent patency [109,111,112].

Post-operative adjuvant PDT also appears to be beneficial for patients with CCA who undergo surgical resection with positive margins. Adjuvant PDT in these cases has been shown to prevent tumor recurrence and improve long-term survival [113,114]. As well, multiple studies have shown the benefit of utilizing neoadjuvant PDT to prevent recurrence and improve surgical candidacy. A phase II study of patients who underwent advanced pCCA resection and were treated with upfront PDT found that all patients achieved R0 resection with no active tumor cells in the surgical samples [115]. Another study of seven patients with initially unresectable disease due to tumor burden or tumor extension into the bile duct underwent 6 weeks of PDT and were then re-evaluated with repeat imaging, and all were found to be surgical candidates. All patients achieved R0 resection with long-term outcomes comparable to initially resectable cohorts [116]. Standardized approaches are needed for positioning PDT in the treatment algorithm for CCA with need for larger studies.

Some centers have utilized PDT in patients awaiting liver transplantation for CCA to maintain locoregional tumor control with reported success [117]. Bile duct segments treated with PDT have not been shown to interfere with surgical anastomosis creation [116]. However, further studies are needed to fully delineate PDT outcomes prior to transplantation. As well, PDT has been studied when used in conjunction with other systemic therapies such as chemotherapy and immunotherapy, and the combination had better overall survival outcomes than monotherapy [106,118,119,120]. An umbrella review found PDT improved survival by approximately 250 days in patients with CCA, increased the 2-year survival rate by 1.65–3.1 times when added to stent placement, and increased the 2-year survival rate by 1.47 times when added to chemotherapy [104]. Overall, neoadjuvant PDT remains experimental at present and has been limited to specialized centers, often as part of clinical trials. Further robust studies are needed to establish the role of PDT in this patient population.

### 3.3. Radiofrequency Ablation (RFA)

RFA utilizes high-energy electromagnetic waves to generate heat in a target tissue, inducing an electrothermal ablation leading to coagulative necrosis, cellular apoptosis, and activation of anti-tumor immune responses. For patients with unresectable CCA, locoregional therapy with RFA through ERCP works by identifying the malignant stricture and moving the flexible RFA probe with electrodes at its tip to the lesion under fluoroscopic guidance. Thermal ablation is typically applied for 1–2 min per treatment area. Concurrent placement of a bile duct stent is also performed at the time of RFA with plastic stents if future RFA sessions are planned or metal stents if there are no further sessions [121].

A meta-analysis of eight studies (three randomized controlled, *n* = 420) found that RFA with stent placement conferred a survival benefit compared to stent placement alone (HR 0.47; 95% CI, 0.34–0.64, *p* = 0.09) [122]. In patients with extrahepatic CCA, the combination of RFA and systemic chemotherapy resulted in a significantly longer median overall survival (17.3 months vs. 8.6 months, *p* = 0.004) compared to chemotherapy alone [123]. No prospective randomized controlled trials have been conducted to compare the outcomes of RFA and PDT to date. A few groups have performed retrospective non-randomized comparisons between the two modalities and have found no significant difference in outcomes [124,125]. In a meta-analysis of patients with unresectable extrahepatic CCA (*n* = 2146 patients) comparing PDT, RFA, and stent only, the pooled survival rate was highest in PDT at 11.9 months compared to 8.1 months with RFA and 6.7 months with the stent only strategy [126]. However, it should be noted that these results are based on pooled data from non-randomized trials which, along with significant heterogeneity, limit study conclusions. In subgroup analysis, a study comparing PDT and RFA in patients undergoing systemic chemotherapy for advanced extrahepatic CCA found those with metastatic disease to have improved overall survival with PDT, while those with non-metastatic disease had improved overall survival with RFA [124]. RFA and PDT have varying advantages in the management of CCA. RFA allows for repermeabilization of occluded metal stents, is more affordable, and avoids photosensitivity. PDT, on the hand, allows for repeated sessions allowing for tumor debulking and can treat peripheral lesions that cannot be reached with RFA [127]. Head-to-head randomized controlled trials comparing PDT and RFA are necessary to understand which patient populations may benefit from these therapies; some groups have even shown a potential synergy between the two [124].

### 3.4. Intraluminal Brachytherapy (ILBT)

ILBT involves insertion of a localized radioactive source within the biliary tract adjacent to the tumor to deliver locoregional therapy. A major benefit of ILBT with localized radiation exposure is that the side effects of systemic therapy are avoided. The use of ILBT to treat CCA was first described in 1981 when placement of iridium-192 wires was used to treat pCCA [128]. The procedure has been described through use of a nasobiliary catheter via ERCP, followed by insertion of the treatment catheter through the nasobiliary catheter. In the early years of ILBT, low-dose rates of brachytherapy with iridium and cesium were used; however, at present, high-dose rate brachytherapy has demonstrated clinical benefit and safety [129].

In comparison to stent placement alone, ILBT has been shown to improve quality of life and prolong survival [130,131,132,133]. A meta-analysis (*n* = 641) found that ILBT compared to stent placement alone had improved mean survival with similar rates of complications [134]. A retrospective study comparing ILBT with iodine-125 seeds and palliative surgery found ILBT to have significantly better overall survival, shorter hospital stay, and lower hospital costs [135]. However, in this study ILBT was performed percutaneously. No comparisons exist between the various locoregional therapies of PDT, RFA, and ILBT. Further, ILBT remains the least available of the three endoscopic locoregional modalities due to the need for nuclear medicine and specialized equipment.

## 4. Applications in Artificial Intelligence (AI)

Recent years have seen major advances in the use of artificial intelligence (AI) and machine learning (ML) for the diagnosis and prognostication of CCA. Numerous studies have utilized AI methods including deep learning and convolutional neural networks (CNNs) for the automated detection of CCA on CT, MRI, and histopathology with modest accuracy [136,137,138,139,140,141,142,143]. Given the inherent diagnostic challenges of CCA, results even with AI and ML methods remain limited in sensitivity and specificity. A systematic review of 50 studies utilizing AI in the diagnosis of CCA compared CT, MRI, trans-abdominal ultrasound, EUS, and cholangioscopy and found the highest accuracy when implementing AI with cholangioscopy [137]. Utilizing CNNs with cholangioscopy images has demonstrated high performance, with reported sensitivity and specificity of 94.7% and 92.1%, respectively [144]. Furthermore, a multicenter study (*n* = 154 patients, 2,388,349 images) utilizing CNNs on cholangioscopy images found improved sensitivity over both ERCP-guided brush cytology and forceps biopsy (0.93% vs. 0.4% and 0.36%, respectively) with comparable specificity [145]. AI methods can also be applied to enhance histopathology analysis and interpretation, with numerous groups showing encouraging early results [139,146,147].

AI methods in conjunction with cholangioscopy and EUS imaging offer an exciting development in an already rapidly expanding field. Further studies will elucidate the optimal algorithms, clinical workflows, and integration strategies necessary to fully harness the diagnostic and prognostic capabilities of AI and ML. As ML models improve in real-time image interpretation, lesion characterization, and decision support, this may further enhance diagnostic accuracy, reduce interoperator variability, and facilitate earlier detection of CCA. However, further work is necessary to validate these models on diverse patient populations and to improve both interuser and interdevice variability. Ultimately, AI-assisted endoscopy has the potential to greatly enhance CCA care towards more personalized, precise, and timely interventions.

## 5. Limitations

While recent advances in endoscopic techniques have significantly expanded the diagnostic and therapeutic options for CCA, several limitations must be acknowledged. The performance of these advanced endoscopic procedures remains highly operator-dependent, with outcomes influenced by experience, technical skill, and procedural volume. This variability can affect both diagnostic yield and procedural safety, making it challenging to reproduce results across different practice settings. Access to specialized equipment such as digital cholangioscopes, high-frequency EUS probes, and intraductal imaging is limited in many regions due to high acquisition and maintenance costs. These resource constraints greatly hinder the widespread adoption of advanced endoscopic techniques. Addressing these barriers will be critical to ensuring that the benefits of these technological advances are realized on a larger scale. Many of the studies on locoregional therapies for CCA are limited due to study size and lack of randomization with potential for unadjusted confounders. This may limit study interpretation and generalizability. Nonetheless, it creates an opportunity for further research in hopes to better understand endoscopic treatment positioning with a standardized approach.

## 6. Conclusions

CCA remains a highly aggressive and devastating disease with challenges in early detection, limited curative options, and poor overall prognosis. Despite this, recent years have witnessed remarkable progress in interventional endoscopic techniques that have transformed the diagnostic and therapeutic landscape of CCA. Endoscopic modalities now play a central role not only in tissue acquisition and staging but also in delivering targeted locoregional therapies and varied options for biliary drainage. The versatility of endoscopic approaches through use of EUS, ERCP, and direct cholangioscopy has allowed for comprehensive care for patients with CCA spanning diagnosis, treatment, and palliation. For patients with unresectable or metastatic disease, photodynamic therapy (PDT), radiofrequency ablation (RFA), and intraluminal brachytherapy (ILBT) have emerged as viable treatment options that are both safe and effective. These interventions offer meaningful improvements in quality of life, symptom relief, and survival for a population historically limited to palliative options.

Looking ahead, the integration of endoscopic innovation with molecular profiling and AI has the potential to usher in a new era of personalized, minimally invasive care for patients with CCA. AI-assisted image analysis with endoscopy, combined with real-time decision support and molecular diagnostics, may enhance diagnostic accuracy, guide targeted therapies, and optimize patient selection. As the field continues to evolve, interdisciplinary collaboration and prospective studies will be critical in validating these technologies and establishing evidence-based pathways for the comprehensive management of patients with CCA.

## Figures and Tables

**Table 1:** Diagnostic Yield of Tissue Acquisition Methods for Cholangiocarcinoma.

Modality	Sensitivity (%)	Specificity (%)	Key References
ERCP-guided brush cytology	26–57	97–100	[24,25,26,27,28,29,30,31]
ERCP-guided forceps biopsy	43–67	90–100	[24,25,26,28,29,30]
EUS-guided fine needle aspiration (FNA)	73–94	88–100	[24,32,33,34,35,36,37]
Peroral cholangioscopy (POC) biopsy	60–90	87–99	[38,39,40]

**Table 2:** Endoscopic Locoregional Therapies for Unresectable Cholangiocarcinoma.

	Photodynamic Therapy (PDT)	Radiofrequency Ablation (RFA)	Intraluminal Brachytherapy (ILBT)
Mechanism	Photosensitizer + light → singlet oxygen → tumor necrosis	High-frequency alternating current → thermal coagulation	High-dose localized radiation delivered via catheter
Delivery Method	ERCP or cholangioscopy	ERCP	ERCP-guided nasobiliary catheter placement
Tumor Types Treated	Mainly pCCA, but also used for dCCA	Both pCCA and dCCA	Mostly pCCA
Survival Benefit	Improves overall survival (HR ~0.52 vs. stent alone)	Improves survival (HR ~0.47 vs. stent alone)	Improves survival vs. stent alone
Adverse Events	Light sensitivity, cholangitis, photosensitivity skin reactions	Cholangitis, hemobilia, pancreatitis	Radiation exposure risks, catheter dislodgement
Limitations	Requires light activation; limited in complex strictures	Limited in tight or angulated strictures	Access and radiation safety; limited availability
Neoadjuvant Use	Shown to increase R0 resection rate in small studies	Limited data	Limited data
Combination with Chemotherapy	Synergistic effect with chemotherapy (e.g., gemcitabine/cisplatin	Shown to improve survival vs. chemotherapy alone in some studies	Studied in combination with EBRT and systemic therapy
Availability	Limited to specialized centers	Increasingly available	Limited to specialized centers

## Abbreviations

The following abbreviations are used in this manuscript:

CCA	Cholangiocarcinoma
iCCA	Intrahepatic Cholangiocarcinoma
pCCA	Perihilar Cholangiocarcinoma
dCCA	Distal Cholangiocarcinoma
PSC	Primary Sclerosing Cholangitis
CT	Computed Tomography
MRI	Magnetic Resonance Imaging
MRCP	Magnetic Resonance Cholangiopancreatography
EUS	Endoscopic Ultrasound
ERCP	Endoscopic Retrograde Cholangiopancreatography
EUS-FNA	Endoscopic Ultrasound–Fine Needle Aspiration
IDUS	Intraductal Ultrasound
pCLE	Probe-based Confocal Laser Endomicroscopy
PDT	Photodynamic Therapy
RFA	Radiofrequency Ablation
ILBT	Intraluminal Brachytherapy
EUS-BD	Endoscopic Ultrasound–Biliary Drainage
SEMS	Self-expandable Metal Stent
AI	Artificial Intelligence
ML	Machine Learning
CNN	Convolutional Neural Network
